# A New Modified Experimental Meibomian Gland Injury Model: Partial Loss of Gland Due to Orifice Cauterization and the Alleviating Potential of 22-Oxacalcitriol

**DOI:** 10.3390/jcm10010006

**Published:** 2020-12-22

**Authors:** Kai Jin, Motoko Kawashima, Masataka Ito, Reiko Arita, Kokoro Sano, Kazuo Tsubota

**Affiliations:** 1 Department of Ophthalmology, Keio University School of Medicine, Tokyo 160-8582, Japan; jinkai_2009@yahoo.co.jp (K.J.); arita.reiko@gmail.com (R.A.); tocotocorody@gmail.com (K.S.); tsubota@z3.keio.jp (K.T.); 2 Department of Developmental Anatomy, National Defense Medical College, Saitama 359-8513, Japan; masataka@ndmc.ac.jp; 3 Tsubota Laboratory, Inc., Tokyo 160-0016, Japan

**Keywords:** meibomian gland, maxacalcitol, atrophy, ductal dilation, injury model

## Abstract

1α,-25-dihydroxy-22-oxacalcitriol (maxacalcitol) is a non-calcemic vitamin D3 analog clinically approved to treat psoriasis, and its role has been increasingly recognized in suppressing keratinocyte proliferation, mediating inflammation, and regulating the immune response. A large number of studies have suggested that vitamin D plays an important role in maintaining ocular surface health. However, its topical effects on the Meibomian gland (MG) has been insufficiently investigated. Here, we introduce an experimental MG orifice injury model, where the partial glandular loss occurred after electrical cauterization on a limited number of MG orifices, and investigate the efficacy and safety of maxacalcitol ointment in treating this MG orifice injury model. We confirm the alleviation of MG atrophy and ductal dilation by maxacalcitol ointment application. The recovery of injured MG visualizing as the residual MG area is significantly better in the maxacalcitol group (*p* = 0.020) compared with the Vaseline^®^ group, especially during the first two weeks. The cornea and other ocular tissues were not affected by maxacalcitol ointment application during our two-month observation period. Altogether, this work indicates that maxacalcitol has therapeutic potential in the amelioration of initial injury of MG orifices caused by electrocautery.

## 1. Introduction

Meibomian glands (MGs) are a special type of sebaceous gland located inside the tarsal plate that excrete lipids (meibum) onto the ocular surface in a holocrine manner. Meibum participates in the composition of the tear film, the first critical refractive interface in the eye. The acinar atrophy and ductal cystic dilatation of MGs are two of the important proposed pathogenetic mechanisms for ocular surface diseases, like Meibomian gland dysfunction or its related evaporative dry eye disease. Patients with those diseases often complain of intermittent/transient blurred vision, pain, or irritative symptoms, thus limiting vision-related quality of life or daily activities [1,2,3]. Among those patients, a large proportion of Meibomian gland “disuse” dropout cases can be found. A recent study regarding the influence of aging on the Meibomian gland indicates that aging-related glandular dropout rather than hyper-keratinization may play a major role in aging or premature mice and clinical Meibomian gland dysfunction [4,5,6,7]. Furthermore, MGs’ glandular loss can often be observed in other cases like Sjögren’s syndrome, ocular graft-versus-host disease, daily contact lens wearers, ocular rosacea, dry eye after lens surgery, and eyelid tattooing [8,9,10,11,12]. Therapeutic strategies for Meibomian gland dysfunction-related dry eye disease includes lid hygiene, topical antibiotics (doxycycline, azithromycin) and steroids, and physical therapies (MG massage, MG probing, LipiFlow^®^, intense pulsed light (IPL) combined with Meibomian gland expression) [13,14,15,16]. Some of the therapies may help release the accumulated meibum and provide symptomatic relief or tear film stabilization. However, treatment for glandular loss is still required because no definitive method is currently available to restore the structure or function of the injured MGs.

Vitamin D has traditionally been recognized to have functions in the intestinal mucosa and bone, where calcium homeostasis is critical for skeletal mineralization. Recently, the roles of its hormonal form 1α,-25-dihydroxy-vitamin D3 and its synthetic analogs have been demonstrated in the health of multiple organ systems. Epidemiological studies demonstrate that vitamin D levels and the genetic variations of vitamin D metabolic components influence a wide range of ocular pathologies like uveitis, myopia, and age-related macular degeneration [17,18,19,20]. However, the link between vitamin D and MG pathology is poorly understood, and the effects of topical 1α,25-dihydroxy-22-oxacalcitriol (maxacalcitol) on MG has not been investigated.

A slightly electrical cauterization can create a murine model on the orifices of MG [21,22] to study the effect of therapeutic agents for rescuing MGs injury-induced glandular loss. Unlike previously reported models, we cauterized only a few orifices (3–4 orifices on the central part of upper eyelid) in this paper. We aim to investigate the recovery process of the glandular “disuse” dropout induced by a slight electrical burn. Here, we introduced an experimental MG injury model where partial glandular “disuse” dropout of MG occurred after a slightly electrical cauterization on a limited number of the MG orifices. Secondary inflammation around the injury site can also be observed in this model. Then, we examined the rescue potential of topical application of maxacalcitol ointment in treating this model.

## 2. Materials and Methods

All study procedures conformed to the principles outlined in the Guide for the Care and Use of Laboratory Animals, published by the USA National Institutes of Health (NIH, 8th edition, published in 2011, ISBN-10: 0309154006, ISBN-13: 978-0309154000). All experiments were approved by the Animal Experimentation Ethics Committee of the Keio University School of Medicine, Tokyo, Japan (permission number 08067) and were conducted following the Association for Research in Vision and Ophthalmology (ARVO) Statement for the Use of Animals in Ophthalmic and Vision Research.

### 2.1. Animals

Six-week-old male C57BL/6JJc1 mice were used in this study. Animals were anesthetized with an intraperitoneal injection of pentobarbital (10 mg/kg) and isoflurane inhalation before the electrocautery (TR4000 Ophthalmic Micro-Diathermy, Medical Instrument Research Associates, Waltham, MA, USA) of four MG orifices in the central one-third of the right upper eyelid. The upper left eyelid was not cauterized so that this eye could serve as the normal control. To standardize the cauterization process, electrocautery was performed by a single examiner on the right eye of all animals using the same energy intensity (slightest burning) and pulse duration (0.5 s). This study was a randomized, contralateral (right eyelid fulgurated, fellow eyelid control), single-blinded trial. The image evaluators did not know which specimens came from cauterized eyelids.

### 2.2. Establishment of an Experimental Meibomian Gland Injury Model and the Treatment with Maxacalcitol Ointment or Vaseline^®^

Two days after cauterization, the study drug was applied to both eyes. The non-cauterized left eyes were used as physiological controls. Mice were randomly divided into two treatment groups with equal numbers of animals in each group (*n* = 10). The treatment group received maxacalcitol ointment (25 µg/g 22-oxacalcitriol [active vitamin D analog], Chugai Pharmaceutical Co., Ltd., Tokyo, Japan) and the treatment control group received Vaseline^®^ ointment (Kozakai Pharmaceutical Co., Ltd., Tokyo, Japan). An average of 4.7 ± 1.2 mg of ointment/dose was used. The ointment was applied to both sides of the upper lid margin once a day, five days a week, for two consecutive months. Animals were euthanized two weeks, four weeks, and two months after treatment commenced, at which point both upper eyelids were harvested for analysis. Five mice in each group were euthanized at two weeks, four weeks, and two months of the lid margin administration. We repeated the experiment three times.

### 2.3. Whole-Mount Tissue Meibography and Analyses

Formalin-fixed eyelids, skin, and subcutaneous tissues were manually removed using fine forceps. Specimens were then bathed overnight in 15%, and then 30% sucrose in PBS (Gibco by Life Technologies, Carlsbad, CA, USA). This process made the connective tissues, including palpebral tissue, transparent, while keeping the lipid-rich MGs opaque. Photographs of the MGs were taken using a dissecting microscope with a transmitted light source and a digital camera (VB-6000, Keyence, Osaka, Japan). Digital MG photographs were then converted into grayscale images using Adobe Photoshop CS4 (Adobe, San Jose, CA, USA). Fulgurated MG orifices appeared black in these images, and the MG orifice surface area was measured using ImageJ 1.49 software (National Institutes of Health, Bethesda, MD, USA).

### 2.4. Histochemical Examination and Immunofluorescent Staining

HE staining was performed on all excised eyelids after they had been fixed in a 10% formalin solution (Wako, Osaka, Japan), embedded in paraffin, and sectioned into 5 µm slices. Slices were stained with filtered hematoxylin for 2–3 min and with eosin for 1–2 min, then rinsed with distilled water several times, dehydrated with 70–100% ethanol in 10% increments, and rinsed with xylene twice for 5 min each time.

Neutral lipids were detected in fresh frozen sections by re-hydrating tissue in 1× phosphate-buffered saline (PBS) and fixing it in 4% paraformaldehyde solution for 30 min. The tissue was then rinsed with distilled water three times for 5 min each and with 60% 2-propanol for 1 min. Next, the tissue was stained with freshly filtered 0.5% Oil Red O (Sigma Chemical Co., St. Louis, MO, USA) for 1 h. A 60% 2-propanol solution was again used to rinse the tissue twice for 5 min each. The hematoxylin stain was applied for 2 min, and the slides were washed in running tap water for 10 min. Finally, the slides were rinsed with distilled water three times and mounted onto glass slides using Mount-Quick “aqueous” mounting medium (Daido Sangyo Co., Ltd., Tokyo, Japan). Eyelids were fixed overnight in 4% paraformaldehyde in 0.01 M PBS (pH, 7.4) and then embedded in OCT, frozen in liquid nitrogen, and sectioned using an HM 550 cryostat (Micro-edge Instruments Co., Tokyo, Japan). Frozen sections (7–8 µm) were placed on glass slides and allowed to dry before staining. Sections were re-hydrated with 0.1% Triton X-100 in 0.01 M PBS three times, and then blocked with 1% BSA in 0.01 M PBS for 30 min at 37 °C. Frozen sections were treated with a 1:200 dilution of rabbit anti-cytokeratin 6 antibody and rat anti-vitamin d receptor antibody (Abcam, Cambridge, UK) at 4 °C overnight; after rinsing three times, sections were treated with Alexa Fluor 488-conjugated anti-rabbit IgG antibody and Alexa Fluor 555-conjugated anti-rat IgG antibody (Molecular Probes, Carlsbad, CA, USA). Cell nuclei were stained with DAPI (Dojindo Laboratories, Kumamoto, Japan). For negative controls, sections were left either un-stained or incubated with irrelevant IgG or secondary antibodies alone. Sections were then evaluated and imaged under a confocal microscope (Axio Imager; Carl Zeiss, Inc., Thornwood, NY, USA).

### 2.5. Statistical Analysis

Student’s *t*-test was used to compare the differences between maxacalcitol group and Vaseline^®^ group data. All statistical analyses were performed with SPSS v.18.0 (IBM Corp., Armonk, NY, USA). Statistical significance was defined as *p* < 0.05.

## 3. Results

### 3.1. Identification of the Glandular Loss (Dropout) Following Meibomian Gland Orifice Cauterization

**Figure 1:**
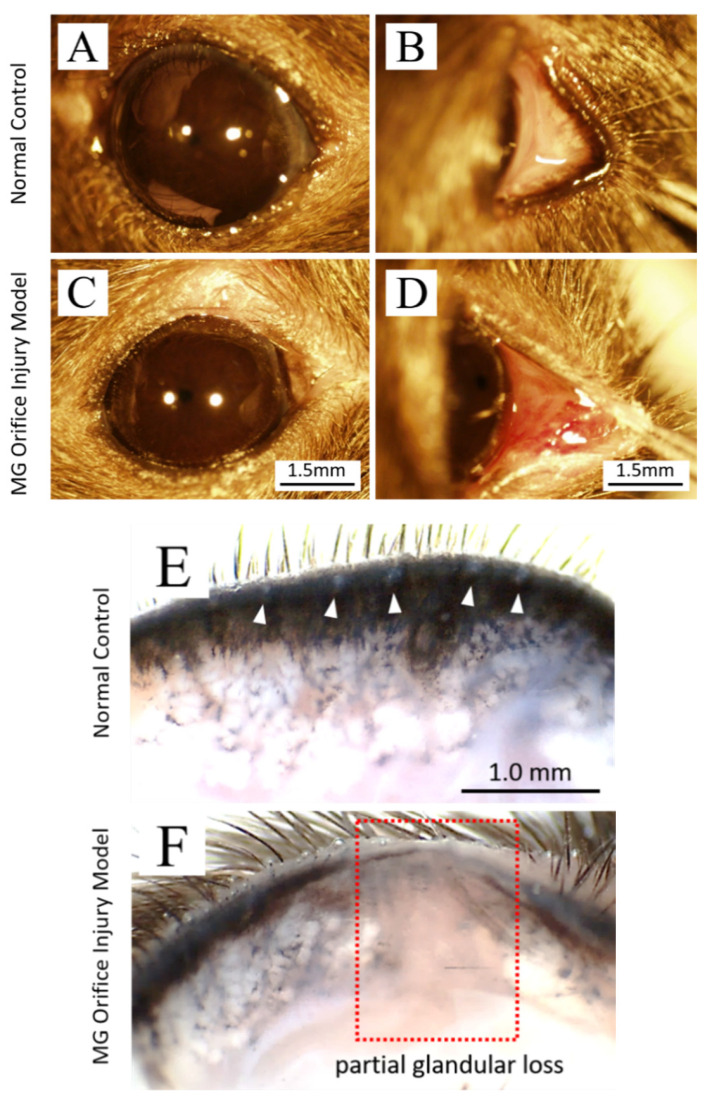
Photographs of the ocular surface and upper eyelid in mice with cauterization-induced Meibomian gland injury and normal eye. (**A**). The ocular surface of normal control eyes is intact, and no damage or discharge is apparent. (**B**,**C**). The conjunctiva shows no signs of congestion, edema, or damage, and one line of round-shape orifices of Meibomian gland can be seen on the lid margin (white arrowhead indicates). (**D**). The ocular surface of an eye with cauterized Meibomian gland orifices appears intact and has no impairment or discharge. (**E**). The upper eyelid conjunctiva in an eye with cauterized orifices shows hyperemia and dropsy. (**F**). Partial glandular loss and lid margin depigmentation in the upper eyelid where orifices were cauterized ((**F**), red dotted line frame). Three or four Meibomian gland orifices in the middle area of upper-right eyelid were cauterized. All experiments were repeated three times and similar results were obtained.

Representative examples of eyes with (right eye) and without (left eye) cauterization-induced MG orifice injury are shown in Figure 1. In eyes that did not undergo orifice cauterization (Figure 1A–C), the ocular surface is intact and shows no apparent damage or discharge, conjunctival congestion, edema, or other signs. In eyes with cauterization-induced MG orifice injury, the ocular surface appears similar to those of eyes without cauterization-induced MG orifice injury (Figure 1D). However, the conjunctival surface was hyperemic and swollen 24 h after cauterization of MG orifice (Figure 1E). Partial MG devitalization/dropout occurred in glands corresponding to the orifices that were electrically cauterized. Control lids had normal, intact MGs that were regularly arranged with no evidence of partial or total glandular loss (Figure 1C). Under microscopy, MGs that had been devitalized/dropout appeared transparent on lid preparations, unlike surrounding intact glands that appeared as grey-white areas (Figure 1F).

### 3.2. Effect of Maxacalcitol on the Recovery of Injured Meibomian Gland

**Figure 2:**
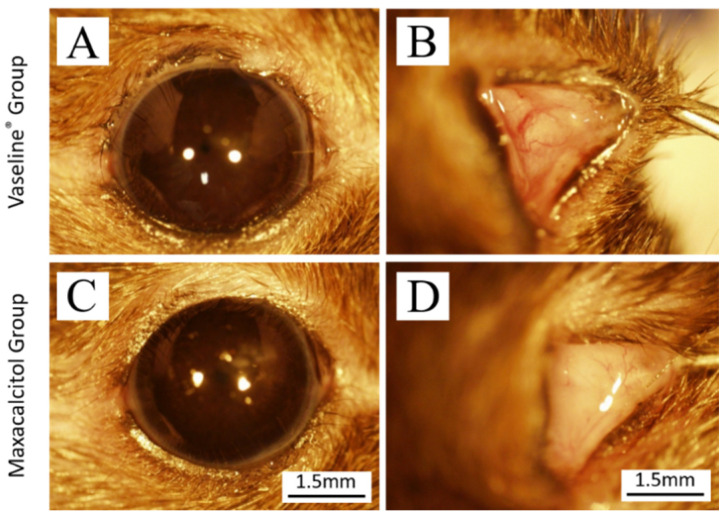
Effect of maxacalcitol ointment and Vaseline^®^ on the ocular surface and conjunctiva. (**A**) After two months of treatment with Vaseline^®^, the ocular surface appears intact and without damage or discharge. (**B**) Slight conjunctival congestion and edema can be seen in the Vaseline^®^ group. (**C**) After two months of treatment with maxacalcitol ointment, the ocular surface appears intact without damage or discharge. (**D**) The conjunctival mucosa appears intact and does not have redness or swelling in the maxacalcitol group. All experiments were repeated three times and similar results were obtained.

Two months into the study treatment, the group of eyes with cauterization-induced MG orifice injury treated with Vaseline^®^ had demonstrated slight conjunctival congestion or swelling (Figure 2B). In contrast, the group of eyes with cauterization-induced MG orifice injury treated with active-type vitamin D3 (maxacalcitol) had intact conjunctival mucosa and no evidence of redness or swelling (Figure 2D). The corneal surfaces in the two treatment groups did not appear different (Figure 2A,C).

The hematoxylin and eosin (HE)-stained eyelid tissues showed that ductal dilatation and acinar atrophy were more severe in the Vaseline^®^ group than in the maxacalcitol group, especially during the first two weeks of treatment. After one week of treatment, a distinctly dilated duct was seen in the Vaseline^®^ group (Figure S1A). After further treatment (2–4 weeks), several dilated cysts were seen, and acini atrophy was apparent (Figure S1B,C). In the maxacalcitol group, a dilated gland was identified, but the duct’s cystic dilation was not as severe as in the Vaseline^®^ group (Figure S1D–F).

**Figure 3:**
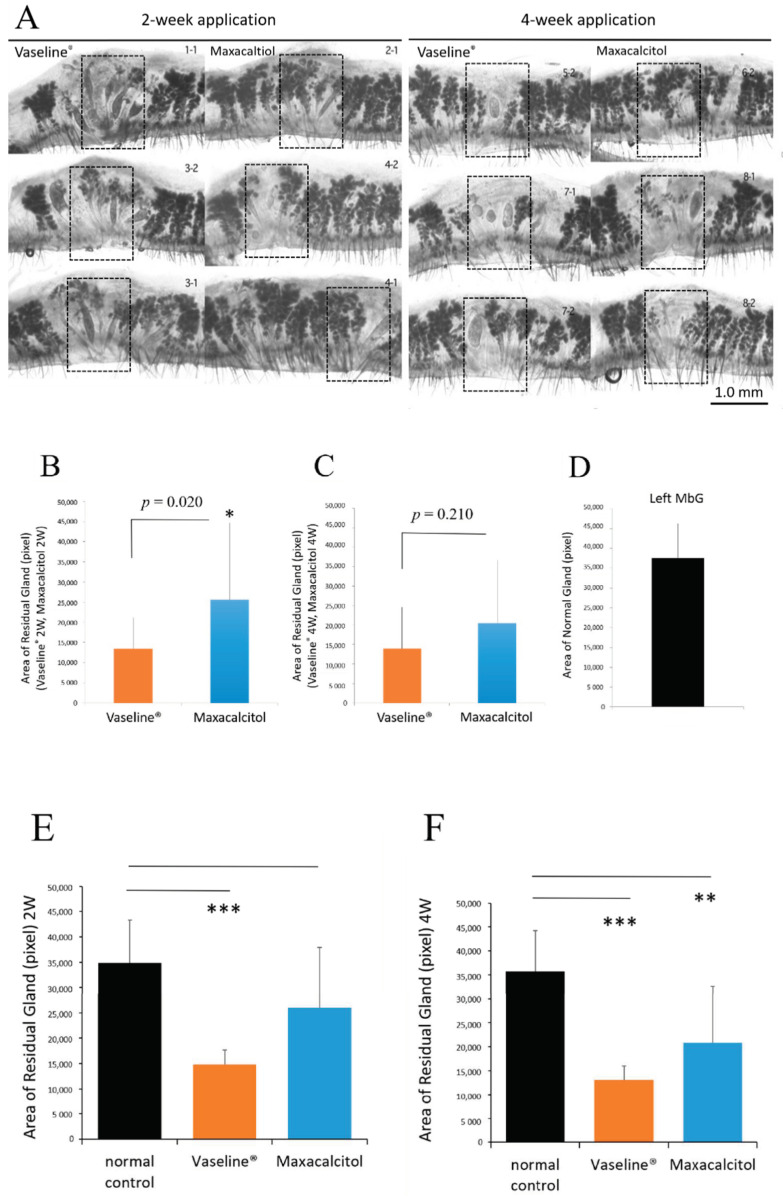
Effect of maxacalcitol ointment and Vaseline^®^ on the Meibomian glands, as visualized with whole-mount Meibography. Make whole-mount Meibography samples of eyelids and observe through a microscope, followed by a comparative analysis of the Meibomian glands’ residual area where 3 or 4 orifices were electrically cauterized (black dotted line frame). (**A**). Microscopic image of a whole-mount eyelid slice made transparent. More distinctly, small secretory acini, cysts, and dilated ducts were present in the Vaseline^®^ group than in the maxacalcitol group. (**B**). The residual area of Meibomian glands was quantified using ImageJ with a grey value adjusted to 128. At least 10 photographs from each group were included in the analysis. After two weeks of treatment, the average residual area was significantly larger in the maxacalcitol group than in the Vaseline^®^ group (*p* = 0.020). This suggests that atrophy and duct plugging were not as severe as in the Vaseline^®^ group. (**C**). After four weeks of treatment, the average residual area remained larger in the maxacalcitol group than in the Vaseline^®^ group. However, it showed no significant difference (*p* = 0.210). (**D**). Image of an un-cauterized eyelid, which served as normal control. (**E**). A statistical difference in “area of residual gland” between the normal control and two-week Vaseline^®^ treatment group was shown (*p* < 0.001), but there was no significant difference between the normal control and two-week maxacalcitol treatment group. (**F**). Statistical differences in “area of residual gland” between the normal control and four-week Vaseline^®^ treatment group (*p* < 0.001) or four-week maxacalcitol treatment group (*p* < 0.01) were shown. All experiments were repeated three times and obtained similar results (*n* = 10). Graphs represent mean ± SEM of three to four independent experiments. Statistical analysis was by Student’s *t*-test, * indicates *p* < 0.05, ** indicates *p* < 0.01, *** indicates *p* < 0.001.

Meibography of whole-mount eyelids showed that more atrophic acini, characterized by small, irregularly-shaped acini (normal acini are round), were present in the MGs of Vaseline^®^-treated eyelids than in those of maxacalcitol-treated eyelids. Additionally, more ductal dilation and cystic formations were observed in the Vaseline^®^ group than in the maxacalcitol group (Figure 3A). After two weeks of treatment, the MG residual area was significantly greater in the maxacalcitol group (*p* = 0.020, Figure 3B). After four weeks of treatment, the residual area was still greater in the maxacalcitol-treated eyelids; however, this difference was no longer statistically significant (*p* = 0.210, Figure 3C). The same area of MGs was also measured in the fellow eye that did not undergo MG orifice cauterization, and is shown for comparison (as normal control) in Figure 3D.

Meibum lipids were visualized using Oil Red O staining. Glandular atrophy and duct plugging were minor after two weeks of maxacalcitol treatment (Figure S2A,C). However, progressive cysts with pooled meibum lipids were observed in both the Vaseline^®^ and maxacalcitol groups after four weeks of treatment (Figure S2B,D).

### 3.3. Adverse Effects on the Cornea and Other Ocular Tissues

**Figure 4:**
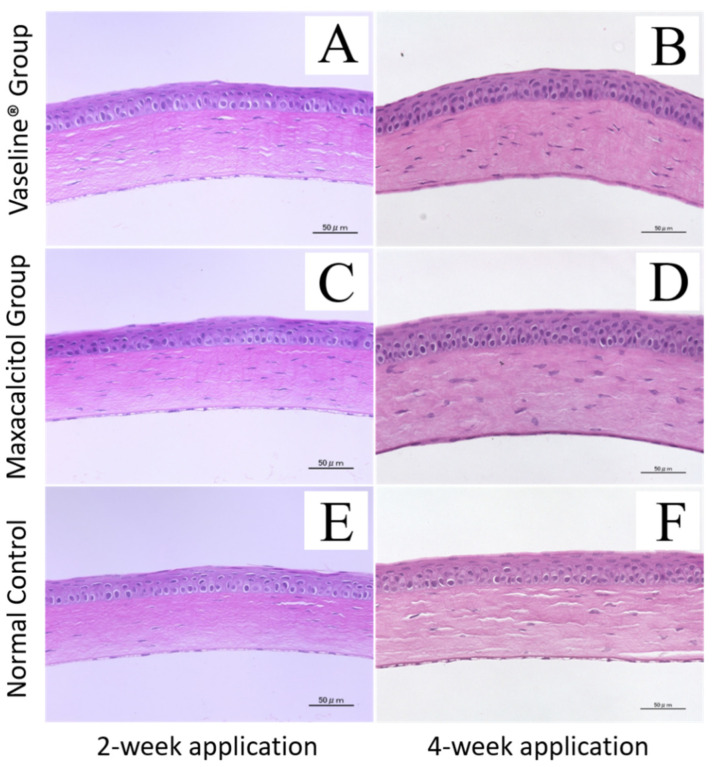
Corneal safety after treatment with maxacalcitol ointment or Vaseline^®^. Microscopy images of paraffin-embedded sections stained with hematoxylin and eosin (HE). All images have a magnification of 400×. Bar, 50 μm (**A**,**B**). After two and four weeks of treatment with Vaseline^®^, all corneal layers appeared normal (**C**). After two weeks of treatment with maxacalcitol ointment, the corneal epithelium and layer thicknesses appeared normal (**D**). After four weeks of treatment with maxacalcitol ointment, the corneal epithelium and layer thicknesses remained normal (**E**,**F**). Image of the cornea of the un-cauterized eye where MG orifices were not electrically cauterized (normal control). All experiments were repeated three times and similar results were obtained.

The safety of relatively long-term maxacalcitol ointment use was determined by examining the cornea’s morphological changes, crystalline lens, and retina, as visualized with HE staining. Even after two and four weeks of Vaseline^®^ or maxacalcitol ointment application, the corneal epithelium and stromal layer depth appeared normal (Figure 4). The crystalline lens’s morphological structures and retina also appeared normal (Figures S3 and S4).

## 4. Discussion

Meibomian gland orifice injury may be caused by many reasons, including physical damage (extreme temperature, laser, radiation, electricity, ocular surgery, long-term exposure to evaporative stress), chemical burns (strong acid or alkali), and physio-pathological insults (chronic inflammation, infection, dysfunctional immune system) [22,23]. Once the Meibomian gland orifices are injured and occluded, the meibum will be retained, which ultimately induces progressive duct dilatation or glandular atrophy/dropout. Previously, Gilbard et al. cauterized all the orifices of white rabbit eyelids and found that ductal dilation and inflammatory infiltration appeared significantly at 12 weeks postoperatively [24]. In a more recent murine model reported by Nichols et al., all the cauterized and stated progressive morphological changes of the orifices (ductal engorgement, cystic formation) happened during the 12 weeks postoperatively [25]. The two reports cauterized all the orifices, mimicking the severe obstructive MGD-like pathology. However, in this study, we cauterized only four orifices of eyelids in each mouse, and the progressive glandular loss was small. Thus, this MG injury model mimics the more common mild-to-moderate MG obstruction cases in clinical practice.

Interestingly, we found that the changes in acinar atrophy and ductal dilatation in our orifice injury model were, to some extent, attenuated by maxacalcitol ointment, especially during the first two weeks of rescue (Figure 4, Figures S1 and S2). The reason might be that maxacalcitol can ameliorate the cicatrization or the wound healing of the electrically cauterized orifice area, leading to terminal duct occlusion. However, it is difficult to figure out whether maxacalcitol accelerates orifice recovery or improves orifice obstruction. It has been reported that in mammary epithelial lineages, active-type vitamin D3 upregulates the gene expression of DPP4/CD26, which has been recently recognized to play pivotal roles in dermal wound healing [26,27]. Therefore, whether maxacalcitol and its metabolites can accelerate wound healing after orifice cauterization and then rescue the atrophy or ductal dilatation needs to be further investigated.

The observed anti-inflammatory activity of maxacalcitol in this partial orifice injury model may offer another clue on VDR ligand efficacy (Figure 2 and Figure 3). It was reported that VDR ligands could suppress ocular surface inflammation and inflammatory changes associated with pancreatitis [18,28]. Our study observed that hyperemia and lid margin swelling were alleviated by topical application of maxacalcitol ointment.

Maxacalcitol ointment is a non-calcemic VDR ligand, and it was able to minimize the risk of hypercalcemia during our experiment. Vitamin D3 interacting with VDR either in the cell nucleus or in the plasma membrane can generate activating responses. Our immunofluorescent results showed that VDR was more likely expressed in the membrane of conjunctival epithelium and ductal epithelium near the orifice (Figure S5), suggesting rapid responses of VDR involved in the maxacalcitol ointment treatment [29]. Furthermore, in our preliminary in vitro experiment using the human Meibomian gland epithelial cell line, the metabolic enzymes of vitamin D3 could inactivate maxacalcitol rapidly in a negative feedback mechanism, thus limiting its efficacy. That can probably explain why the effect of the relatively long-term four-week treatment was not as good as the effect of two-week treatment in rescuing acinar atrophy or ductal dilatation, followed by orifice injury.

In conclusion, we successfully induced a Meibomian gland orifice injury model to study the secondary glandular dropout and introduced a useful whole-mount meibography approach to study MG atrophy/dropout semi-quantitatively. Our data also supports the efficacy of maxacalcitol ointment in facilitating recovery of injured orifices of the Meibomian gland. Given that topical maxacalcitol ointment did not adversely affect the cornea or other ocular tissues during our observation, further laboratory and clinical studies should be performed to better understand the therapeutic uses of VDR ligands or their metabolites in treating the initial injury of MG orifices and secondary glandular dropout.

## Supplementary Materials

The following are available online at https://www.mdpi.com/2077-0383/10/1/6/s1, Figure S1: Effect of maxacalcitol ointment and Vaseline^®^ on pathological changes associated with electric cauterization-induced Meibomian gland injury, Figure S2: Microscope photographs of meibum lipid, as visualized with Oil Red O staining, Figure S3: The effects of maxacalcitol ointment and Vaseline^®^ on the crystalline lens, Figure S4: The effect of maxacalcitol ointment and Vaseline^®^ on the retina, Figure S5: Mouse conjunctival cells and Meibomian gland epithelial cells express the vitamin D receptor (VDR).
