# Supplementary material for: A New Modified Experimental Meibomian Gland Injury Model: Partial Loss of Gland Due to Orifice Cauterization and the Alleviating Potential of 22-Oxacalcitriol
Source: J Clin Med. 2020 Dec 22;10(1):6. doi: 10.3390/jcm10010006 (PMC7792963; doi:10.3390/jcm10010006)
Supplement: Supplementary file 1 [file jcm-10-00006-s001.pdf]

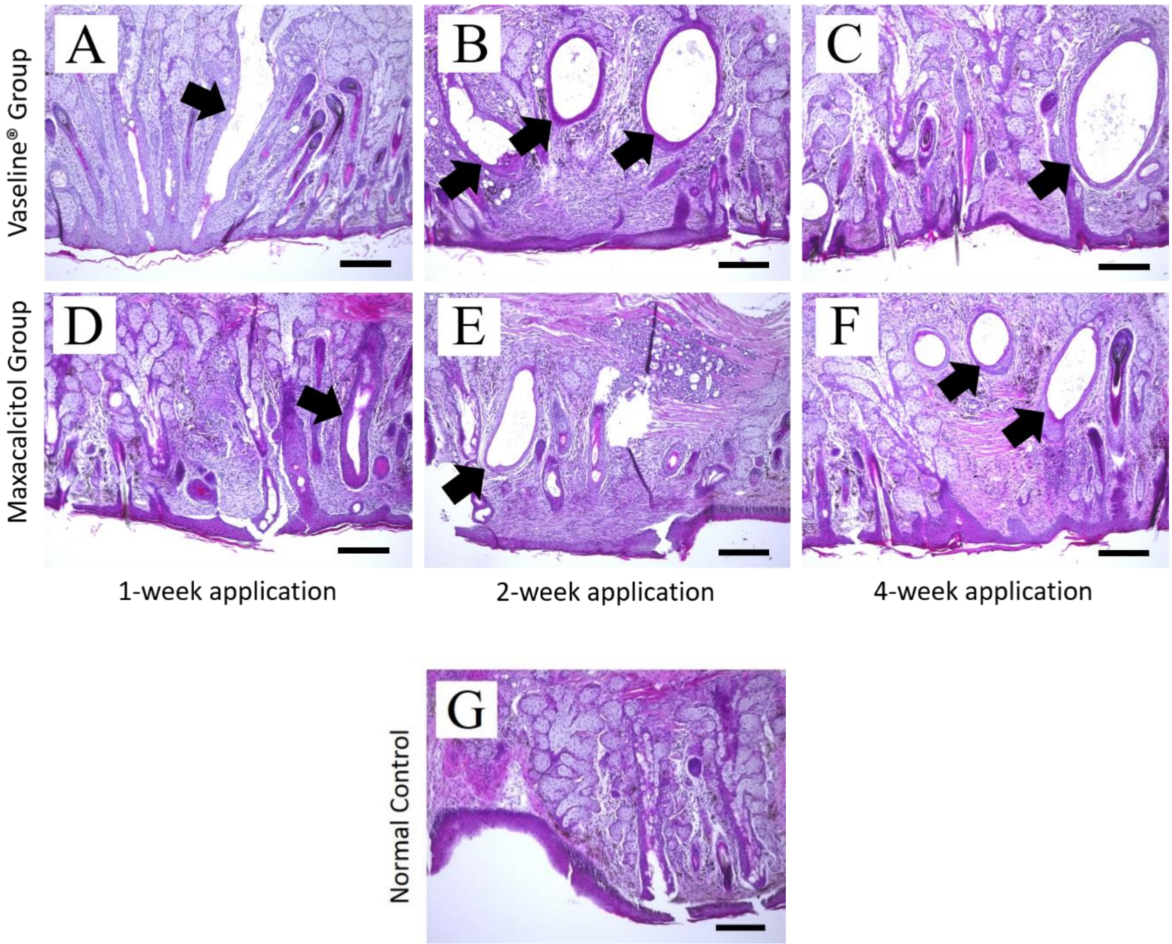

Figure S1. Effect of maxacalcitol ointment and Vaseline® on pathological changes associated with electric cauterization-induced Meibomian gland injury. Microscope photographs of paraffin-embedded lid sections stained with hematoxylin and eosin (HE) are shown. All images have a magnification of 100x. The scale bar represents 200 μm. **A.** The Meibomian gland duct near the orifice was narrowed, while it was dilated in other parts (solid black arrow). **B.** Several central ducts were dilated (solid black arrows) and gradually became cysts accompanied by acinar loss near the cauterization area. **C.** The dilatant cysts became larger in the Vaseline® group. **D.** Dilated ducts of cauterized Meibomian gland orifices were also observed in the maxacalcitol group. **E.** After two weeks of treatment, dilated cysts were smaller in the maxacalcitol group than in the Vaseline® group. Atrophic acini were still present. **F.** After four weeks of treatment with maxacalcitol, dilated cysts were still present. G. In un-cauterized eyelids, the shapes of the acini and Meibomian gland ducts were normal. All experiments were repeated three times and obtained similar results.

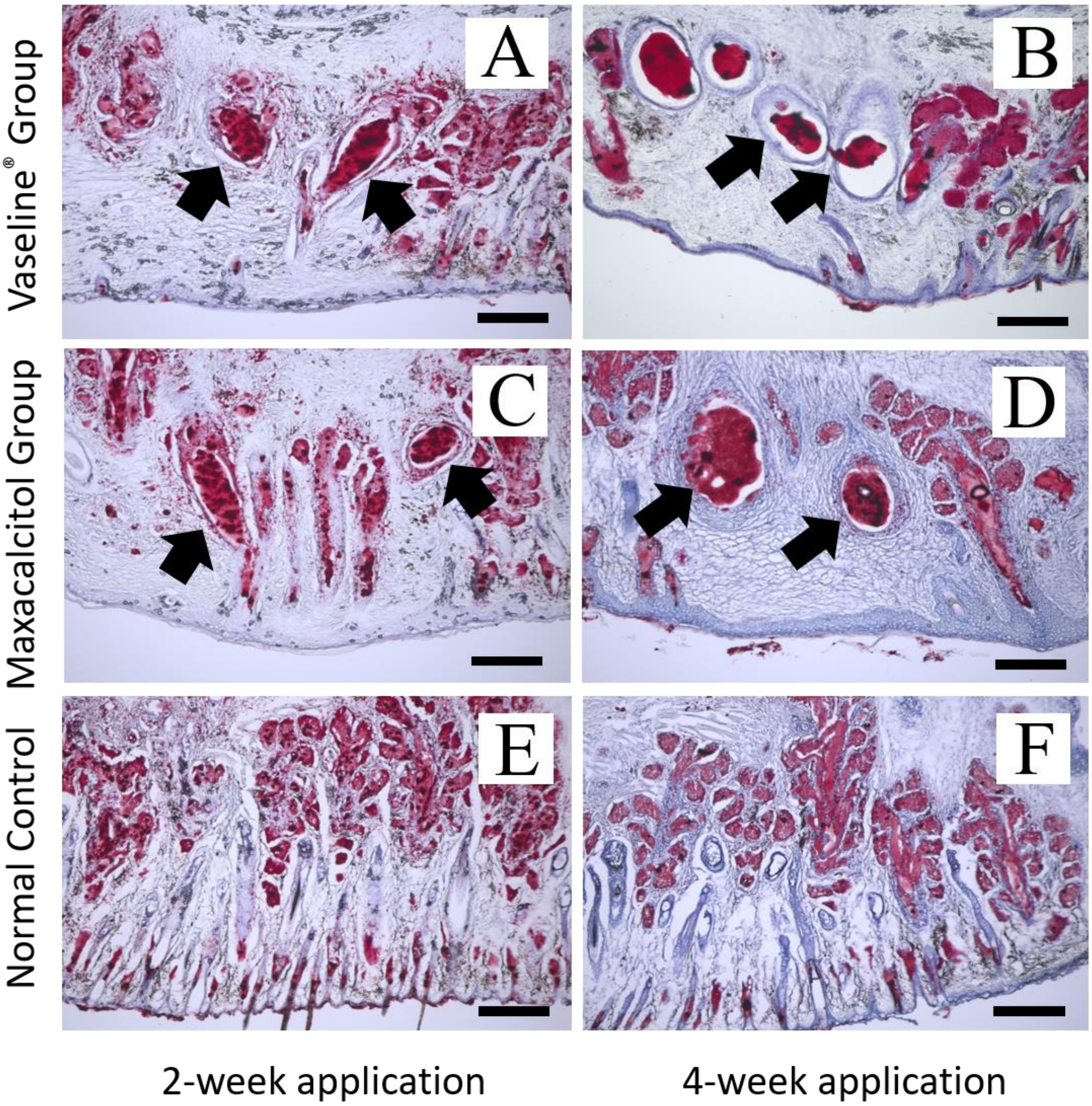

Figure S2. Microscope photographs of meibum lipid, as visualized with Oil Red O staining. Frozen sections were made from the upper eyelids from the Vaseline® group, maxacalcitol group, and normal control, and then stained with Oil Red O to highlight the meibum lipid of Meibomian glands. All images have a magnification of 100×. Bar, 200 μm. **A.** Dilated ducts and atrophic acini were seen. Ducts were narrowed near the orifice (lower) side and dilated on the upper side, where the excreted meibum lipid is deposited (solid black arrows). **B.** Cysts with pooled meibum lipid were seen in a cauterized, but not a normal area. **C.** After two weeks of treatment with maxacalcitol ointment, atrophic acini and dilated ducts are still present. However, several excretory ducts are visible in the cauterized area. This suggests that atrophy and duct plugging were not as severe as in the Vaseline® group. **D.** After four weeks of treatment with maxacalcitol ointment, dilated cysts with pooled meibum lipid were still present. These developed from the excretory ducts after the cauterization of corresponding duct orifices. **E–F.** In un-cauterized eyelids, meibum lipid content, acini shape, and Meibomian gland ducts appeared normal. All experiments were repeated three times and obtained similar results.

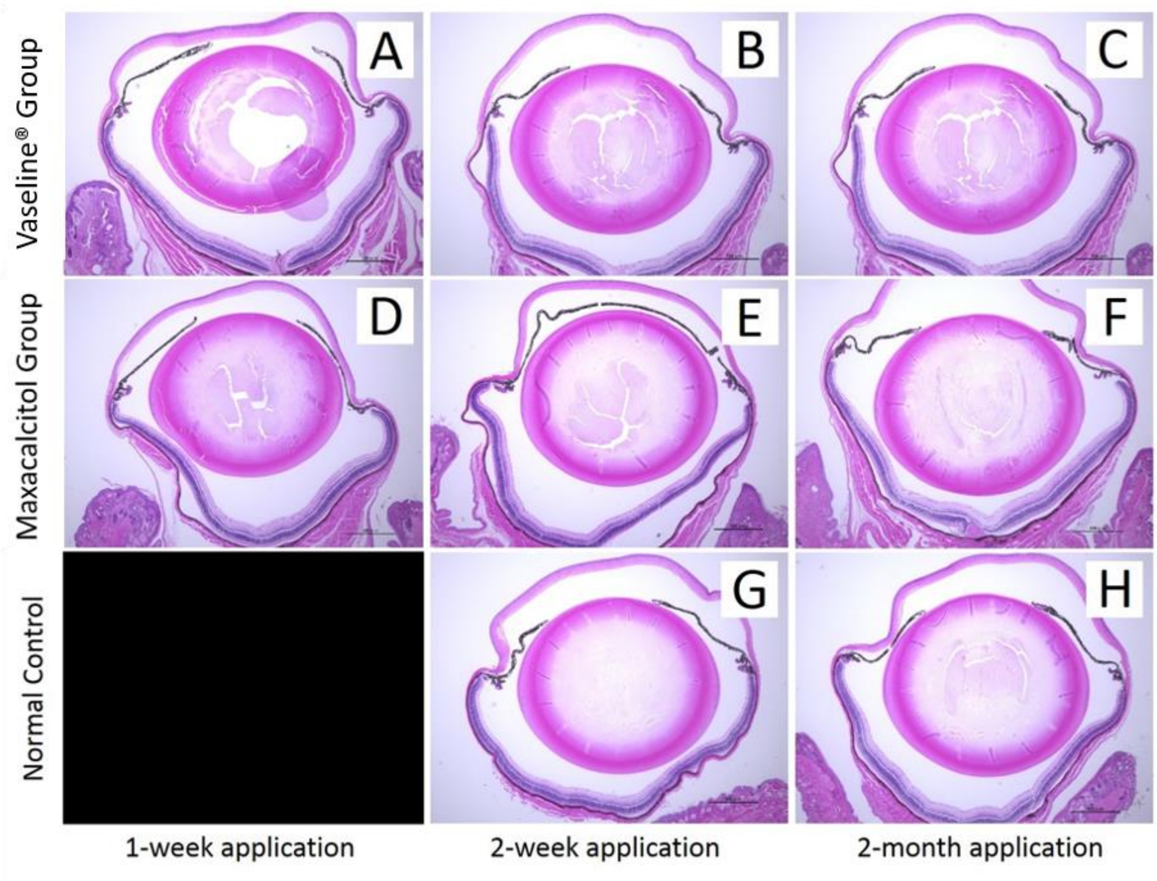

Figure S3. The effects of maxacalcitol ointment and Vaseline® on the crystalline lens. Microscopy images of paraffin-embedded sections were stained with hematoxylin and eosin (HE). All images have a magnification of 40×. **A–H.** The shapes of the lenses in all groups appear normal, the lens fiber layer is not distorted, and abnormal extracellular spaces are not observed in any group during the two-month application of maxacalcitol or Vaseline®. Scale bar, 500 μm. All experiments were repeated three times and obtained similar results.

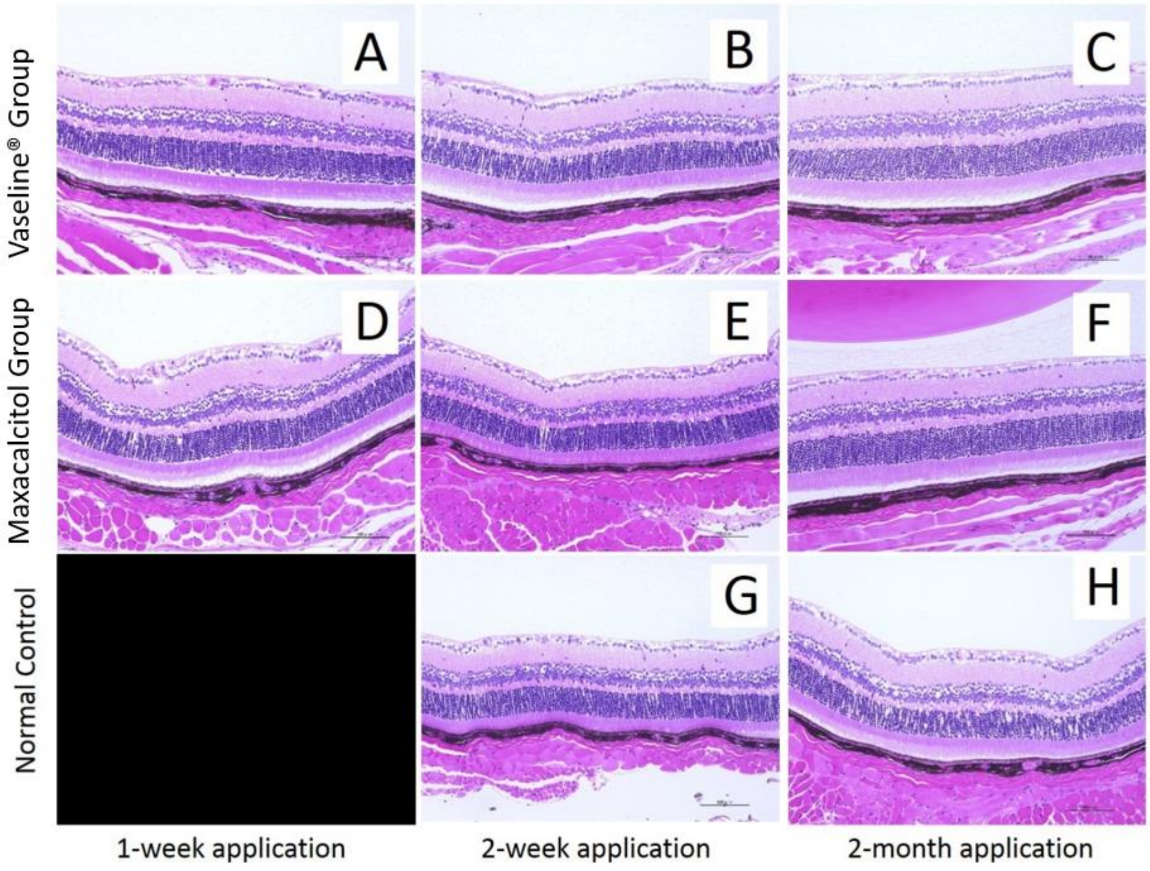

Figure S4. The effect of maxacalcitol ointment and Vaseline® on the retina. Microscopy images of paraffin-embedded sections were stained with hematoxylin and eosin (HE). All images have a magnification of 200×. **A–H.** The retinas' shapes in all groups appear normal, and abnormal changes were not observed in any group during the two-month application of maxacalcitol or Vaseline®. Scale bar, 100µm. All experiments were repeated three times and obtained similar results.

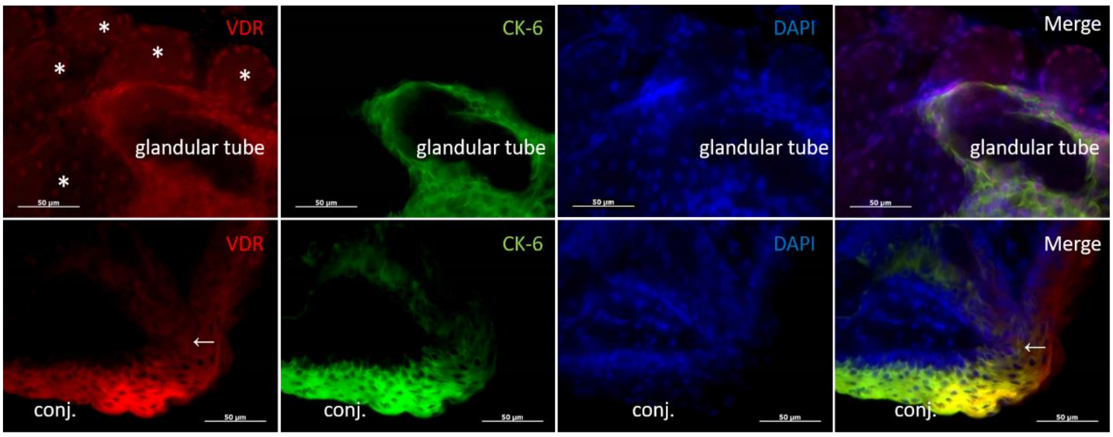

Figure S5. Mouse conjunctival cells and Meibomian gland epithelial cells express the vitamin D receptor (VDR). A. The cryosection of mouse eyelid tissue was fixed and stained for VDR (red), cytokeratin 6 (CK-6, green), and DAPI nuclear stain (blue). The expression of VDR can be observed in the nuclear of acini (*) and glandular tube of Meibomian gland and in the orifice (←) of Meibomian gland (upper eyelid, WT, 400×).
